# The Pattern of Expression of Human Placental Lactogen Across Normal, Lactational, and Malignant Mammary Epithelium

**DOI:** 10.7759/cureus.35125

**Published:** 2023-02-17

**Authors:** Raja S Alyusuf, Javed F Wazir, Urmil P Brahmi, Abdul Rahman E Fakhro, Zainab A Toorani, Yousef Rezk

**Affiliations:** 1 Pathology, Salmaniya Medical Complex, Manama, BHR; 2 Pathology, Royal College of Surgeons in Ireland - Bahrain, Muharraq, BHR; 3 Pathology, St. Peter’s Hospital, Lyne, GBR; 4 Pathology, Arabian Gulf University, Manama, BHR; 5 Surgery, Fakhro Medical City, Manama, BHR; 6 Pathology, The Royal College of Surgeons in Ireland, Muharraq, BHR; 7 College of Medicine, Royal College of Surgeons in Ireland - Bahrain, Muharraq, BHR

**Keywords:** prognosis, immunoexpression, lymph node, tumor, breast, human placental lactogen

## Abstract

The immunoexpression of human placental lactogen (hPL) in mammary epithelium is not well studied in the literature. Our overall objective was to delineate the distribution pattern of hPL across mammary epithelia of varying levels of differentiation. This is the first research to study the level of expression of hPL in human lactational change epithelium. Immunohistochemistry (IHC) for hPL was performed on archival formalin-fixed paraffin-embedded tissue blocks of 97 cases. These consisted of 53 invasive ductal carcinomas, 21 lactational change cases, and 23 cases of normal mammary tissue. The results of this study show underexpression of hPL in malignant epithelium compared to normal and lactational groups individually and combined as a non-malignant group. However, a higher expression of hPL was noted in mammary carcinoma of axillary lymph node (ALN)-positive patients compared to ALN-negative cases. There was no statistically significant difference between hPL expression and tumor grade, estrogen receptors (ER), progesterone receptors (PR), or human epidermal growth factor receptor 2 (HER2) status. The comparison of the immunoexpression of hPL in malignant epithelium versus lactational change epithelium may provide the basis for future studies on the possible role of hPL in the protective mechanism of lactation tissue from carcinogenesis. Our results could be explained by the proposed mechanism in the literature, which is that breast cancer cells have a potential inhibitory effect on the translation of human chorionic somatotropin hormone (CSH) mRNA into hPL protein. Our results support the literature findings of a poorer prognostic outcome for breast malignancies when hPL is expressed but require further studies using a more comprehensive range of clinical parameters.

## Introduction

Human placental lactogen (hPL), also known as human chorionic somatotropin hormone (CSH) [1], is the primary secretory product of the syncytiotrophoblast of the placenta. This peptide hormone is one of the three known human lactogens, along with human prolactin (hPRL) and human growth hormone (hGH) [2-4]. The family of lactogens share a common role of binding to and activating the prolactin receptor (PRLR) [2,4]. Although it has a mere 23% amino acid sequence homology to hPRL in comparison to a higher 85% structural homology to hGH, hPL binds to PRLR with a greater affinity than the growth hormone receptor (GHR) [4]. Additionally, hPL is encoded by the CSH gene, which is a part of the growth hormone (GH) gene cluster on chromosome 17 [2]. Studies have shown that the primary function of hPL is to affect the maternal secretion of insulin and raise the glucose availability to the fetus by causing a reduction in the maternal fatty acid stores [3,5]. 

Furthermore, hPL levels in the maternal circulation peak during mid to late pregnancy. hPL levels are the highest amongst all other protein hormones by the end of gestation [2,6]. A study has shown that lactogenesis occurs during the twelfth to sixteenth week of pregnancy [7], and high hPL levels play a role in a woman’s ability to lactate [1,4,5,8,9]. Given that lactation has been confirmed to be protective against breast cancer [1,10], we note that it was intriguing to explore the existence of a potential effect of hPL on breast malignancy. Accordingly, we have conducted this study to delineate the distribution pattern of hPL in malignant versus non-malignant epithelium, with particular emphasis on the lactational epithelium.

This article was previously presented as an oral presentation at the "Cancer Virtual 2020" webinar held on September 14, 2020.

## Materials and methods

Study design

A retrospective study was performed on archival formalin-fixed paraffin-embedded tissue blocks retrieved from files of the Histopathology Department, Salmaniya Medical Complex, Kingdom of Bahrain.

Data collection

A total of 97 cases were included in this study. This comprised 53 malignant epithelia as follows: 42 invasive ductal carcinomas, two infiltrating duct carcinomas, one invasive mucinous carcinoma, one invasive lobular carcinoma, one mixed lobular and ductal carcinoma, one atypical medullary carcinoma, one invasive ductolobular carcinoma, one mixed ductal and mucinous carcinoma, one invasive micropapillary carcinoma, one residual ductal carcinoma, and one invasive lobular carcinoma solid variant. They ranged between Grades II and III. Furthermore, 21 cases with lactational change and 23 cases showing normal mammary tissue histology were also included. The latter group was taken from excisions performed for benign pathology. Benign lesions seen included abscesses, granulomatous mastitis, reduction mammoplasty, accessory breast, intraductal papilloma, fat necrosis, hyperplasia, and fibroadenoma. All cases were received as routine diagnostic laboratory specimens between 2001 and 2007.

Procedure

Immunohistochemistry (IHC) for placental lactogen antibody Dako Cytomation (Code A0137) (Dako, Glostrup, Denmark) was carried out as follows: Five µm sections were cut and mounted on silane-coated slides, dried, deparaffinized in xylene, and rehydrated in alcohol. Endogenous peroxidase was quenched by 3% Hydrogen peroxidase for 10 minutes. Pre-treatment of tissue with proteolytic enzyme was not required. Slides were incubated overnight at 40℃ with polyclonal placental lactogen antibody Dako Cytomation (Code A0137) in 1:1000 dilution. Immunoreaction was detected and visualized by using a Dako Cytomation LSAB2 System (HRP-code K0673). Positive cases were determined by exhibiting a moderate- to dark-brown cytoplasmic staining pattern in 10% or more cells.

Data analysis 

Other parameters were extracted from the pathology reports of the cases, such as tumor grade, estrogen receptor (ER), progesterone receptor (PR), HER-2 expression, and axillary lymph node (ALN) status. IHC for ER and PR were determined by the Histoscore method (H score). Positivity ranged from 20/300 to 300/300. HER-2 was determined by scoring membranous staining as negative (0/1+), equivocal (2+), or positive (3+). Expression of hPL was compared between malignant and lactational groups, in addition to a comparison between malignant and non-malignant groups. Within the malignant group, hPL expression was further analyzed according to grade, ER, PR, HER-2 and ALN status. Analysis of hPL expression according to HER2 status excluded cases that showed HER2-equivocal results by IHC. Data were analyzed by using the Statistical Package for the Social Sciences (SPSS), version 15 (SPSS Inc., Chicago, USA). Data were grouped into categories and analyzed for correlations using Pearson’s Chi-Square test.

Ethics statement 

The use of human tissue samples was approved by the Salmaniya Medical Complex Ethics Committee.

## Results

There were 19 hPL-positive cases out of 20 normal mammary epithelia (95%) and 20 hPL- positive cases out of 20 lactational change epithelia (100%). Meanwhile, only 29 out of 52 malignant mammary epithelia (55.8%) were hPL positive. Thus, the immunoexpression of hPL among the normal and lactational epithelia groups was significantly higher than that of the malignant epithelium group, with p-values of 0.001 and 0.0001, respectively (Tables 1, 2). However, there was no statistically significant difference between hPL positivity in normal versus lactational epithelia (p=1.00).

**Table 1:** Immunoexpression of hPL in malignant versus normal mammary epithelium *hPL: human placental lactogen **Level of statistical significance is p-value less than 0.05 ***52 malignant mammary epithelium cases included out of a total of 53 due to inadequate staining of 1 ****20 normal mammary epithelium cases included out of a total of 23 due to inadequate staining of 3

	hPL* status				
Category	Positive	Negative	Total	% positivity	p-value**
Malignant	29	23	52***	55.8%	0.001
Normal	19	1	20****	95.0%	

**Table 2:** Immunoexpression of hPL in malignant versus lactational mammary epithelium *20 lactational mammary epithelium cases included out of a total of 21 due to inadequate staining of 1 **hPL: human placental lactogen

	hPL** status				
Category	Positive	Negative	Total	% positivity	p-value
Malignant	29	23	52	55.8%	0.0001
Lactational	20	0	20*	100.0%	

Statistical significance was still achieved after combining the total number of normal and lactational mammary epithelia in the non-malignant group and comparing it with the malignant group (p=0.018) (Table 3).

**Table 3:** Immunoexpression of hPL in malignant versus non-malignant epithelium *40 non-malignant mammary epithelium cases out of a total of 44 due to inadequate staining of 4 **hPL: human placental lactogen

	hPL** status				
Category	Positive	Negative	Total	% positivity	p-value
Malignant	29	23	52	55.8%	0.018
Non-malignant	39	1	40*	97.9%	

Furthermore, a positive correlation was established between hPL expression and the ALN involvement group as 70.6% of the ALN positive cases were hPL positive compared to only 28.6% of the ALN negative cases being hPL positive (p-value= 0.007) (Table 4).

**Table 4:** Immunoexpression of hPL in the malignant mammary epithelium according to ALN status *ALN= Axillary Lymph Node ***hPL: human placental lactogen **4 of the 52 adequately stained malignant mammary epithelium cases were not applicable for immunohistochemistry

	hPL*** status				
Category	Positive	Negative	Total	%hPL Positivity	p-value
Positive ALN*	24	10	34	70.6%	0.007
Negative ALN	4	10	14	28.6%	
Total	28	20	48**		
% ALN Positivity	85.7%	50.0%			

There was no statistically significant difference for hPL positivity in malignant epithelium classified according to tumor grade, ER, PR, or HER2 (p=0.444, 0.390, 0.493, and 0.273, respectively).

## Discussion

Studies have acknowledged that full-term pregnancy decreases the risk of breast cancer [1,11-13]. Due to the presence of significantly high hPL levels towards the end of gestation [2,4,6], studies have suggested that hPL contributed to this protective effect [1,14].

Amidst the scarcity of studies on the extent of the role of hPL in lactation [15,16] and the effect of hPL on breast cancer, we have conducted this research to further delineate the role of hPL along the spectrum of lactation, normal, and malignant mammary epithelium through its immunohistochemical expression. 

A previous study has confirmed the absence of a statistically significant difference in hPL protein expression between mammary carcinomas and benign mammary disease [17]. Nevertheless, other studies have shown that immunoreactive and serum hPL were identified exclusively in patients with breast carcinomas and that none were detected in benign or normal cases [4,18,19], which led to a proposal that hPL could potentially serve as a tumor biomarker for breast cancer [6]. This differed from our results, as we have found that the immunoexpression of hPL in non-malignant cases was significantly higher than that in malignant mammary epithelium. A recently conducted experiment by Tuttle, et al. criticized the methods used by past research to determine the presence of hPL in breast cancer. They highlighted that older methods depended on CSH mRNA levels as surrogates for hPL protein and hPL protein expression, which is inaccurate as the gene was only sometimes translated. Additionally, their experiment signified that older studies reporting hPL expression in breast cancer used non-specific antibodies, which resulted in a misleading conclusion [2]. 

While Kizilgul, et al. reported the presence of immunoreactive hPL in some breast malignancies, their study highlighted the absence of hPL in the serum of some patients with breast cancer [4]. In agreement with this, previous research papers have also portrayed the absence of hPL in breast cancer patients [20], with one study confirming that out of 54 infiltrating ductal breast carcinomas, none contained hPL [21]. This is partly in line with our findings that hPL was not always expressed in malignant mammary epithelium. Our detection of hPL in 55.8% of malignant epithelium could be explained by the intriguing proposal presented in the literature. This explains a possible post-transcriptional inhibitory mechanism of breast cancer cells on the translation of CSH mRNA into hPL protein when the gene is not highly expressed [2].

A study underlined that the presence of hPL in breast tumors indicated a poorer prognosis [2]. A further study also reported that 16 out of 26 patients with breast carcinoma had detectable immunoreactive hPL, and 77% of the tumors with amplified hPL genes had lymph node metastasis. This could serve as a prognostic factor associated with aggressive breast malignancies [6,22]. Additionally, a further study confirmed the presence of a greater incidence of lymph node metastasis with hPL gene amplification, which supports the notion that hPL gene amplification is associated with poor prognosis in breast malignancies [4,8,18].

Furthermore, studies have illustrated an essential connection between PRLR and the progression of breast tumors. Increased membrane ruffling, cell motility, and cytoskeletal changes were consequences of PRLR activation in breast cancer cells. All of these events were correlated with breast cancer progression [8]. Moreover, using L-dopa or ergot compounds to inhibit prolactin has been observed to achieve bone relief in women with metastatic breast cancer, which increases the possibility of prolactin-promoting breast cancer [23]. Additionally, a study has confirmed the possibility of heterodimerization of GHRs and PRLRs in humans [17,24]. This mechanism explained the increased probability of hPL binding to these hybrid receptors and promoting tumor growth in breast cancer cells [4,25]. The positive correlation we have established between hPL expression and ALN involvement may support a poor prognostic outcome, as there was a statistically significant difference (p=0.007) between hPL expression in positive ALN cases compared to negative ALN cases.

Tuttle, et al. and Kizilgul, et al. reported HER2 oncogene amplification with the amplification of CSH genes [2,4]. This is, however, inconsistent with the results we found as there was no statistically significant difference (p=0.273) between hPL positivity in HER2 positive and negative samples. A further study solidified the positive correlation between HER2 positivity and hPL protein expression. It also presented results in line with our findings, and no significant correlation was present between hPL expression and PR or ER presence [17].

Recently, studies on hPL have focused on its expression in placental site trophoblastic tumors (PSTTs), and several papers have established a strong expression of hPL in PSTTs [4,26,27]. A recent study on hPL has discussed the use of immunohistochemistry for hPL as a diagnostic method for PSTT with about 60% specificity [26]. Moreover, researchers have shed light on a rare form of breast neoplasms having a choriocarcinoma differentiation. In this uncommon variant of breast carcinoma, malignant cells similar to chorionic trophoblastic cells react with hPL and human chorionic gonadotropin (hCG) immunohistochemically [4,28,29].

To the best of our knowledge, this is the first study that compares the expression pattern across the lactational epithelium. However, a critique of our study is that a small number of samples were included. Only 20 lactational epithelium cases were presented. Future studies should expand the pool of evidence on the distribution pattern of lactational epithelium. More research should be conducted to test the hypothesis on the inhibitory mechanism of breast cancer cells on hPL expression and the immunoexpression of hPL in mammary carcinoma with syncytiotrophoblastic differentiation.

After reviewing the literature, we recognized the lack of recent evidence concerning the link between breast cancer and hPL. Any mention of such a link was minor and solely based on the available old references. Considering all of the aforementioned, it is crucial to study hPL expression across breast epithelium of varying levels of differentiation, particularly lactational epithelium. However, including lactational epithelium in any histologic-based study is challenging. Retrieving lactational change cases from archival material in a routine diagnostic histopathology laboratory via SNOMED code is not usually possible, as it is not routine practice for pathologists to SNOMED code them. Thus, our study is unique in that we recorded all cases of lactational change from our routine practice in a prospective manner over time in order to conduct this study.

## Conclusions

hPL was under-expressed in malignant mammary epithelium compared to normal and lactational mammary epithelia. This may provide a basis for future research on the potential protective role that hPL plays against breast carcinogenesis, particularly in the lactational epithelium. Our findings may otherwise be explained by the possible inhibitory mechanism that breast cancer cells exhibit on the CSH gene, which suggests that the CSH mRNA is not translated to hPL protein when the gene is not highly expressed. Our results could also dispute the proposed hypothesis of using hPL as a tumor biomarker for breast malignancy as it was under-expressed in the malignant group compared to non-malignant cases. The expression of hPL protein is positively correlated with positive ALN status, which may support the association between hPL expression and a poorer prognostic outcome of breast malignancies. Further studies are needed to confirm this link using established clinical parameters in larger population groups.
